# The Joint Observation in Neonatology and Neurodevelopmental Outcome of Preterm Infants at Six Months Corrected Age: Secondary Outcome Data from a Randomised Controlled Trial

**DOI:** 10.3390/children9091380

**Published:** 2022-09-13

**Authors:** Oriane Lovey, Myriam Bickle-Graz, Mathilde Morisod Harari, Antje Horsch, Juliane Schneider

**Affiliations:** 1 Medicine School, University of Lausanne, 1015 Lausanne, Switzerland; 2 Department of Woman-Mother-Child, Clinic of Neonatology, Lausanne University Hospital and University of Lausanne, Avenue Pierre-Decker 2, 1011 Lausanne, Switzerland; 3 Department of Psychiatry, Division of Child and Adolescent Psychiatry, Lausanne University Hospital and University of Lausanne, 1011 Lausanne, Switzerland; 4 Institute of Higher Education and Research in Healthcare, University of Lausanne, 1010 Lausanne, Switzerland

**Keywords:** developmental care, early intervention, neurodevelopment, preterm neonate, NICU

## Abstract

This study aimed to evaluate the impact of a standardised *joint observation* (JOIN) performed in the neonatal intensive care unit (NICU) on the neurodevelopment of preterm infants at six months corrected age (CA) compared with a preterm control group. In this monocentric interventional randomised controlled trial, we allocated 76 mothers and their preterm neonates to either JOIN, an early one-session intervention, or standard care during the NICU hospitalisation. The neurodevelopment of the preterm infants was assessed by standardised developmental tests at six months CA and compared between the intervention and the control groups. This randomised controlled trial was registered on clinicaltrials.gov (NCT02736136) in April 2016. Sixty-five infants underwent neurodevelopmental assessment at six months CA. There were no significant differences between the two groups in neurodevelopmental outcome measures. The JOIN intervention was not associated with significant improvement in neurodevelopment at six months CA in preterm infants.

## 1. Introduction

Prematurity is one of the most commonly used health indicators in developed countries and the first cause of neonatal death in the world [1,2]. Although advances in antenatal and postnatal care have improved survival and outcomes, preterm neonates still experience higher long-term morbidity than term infants [1]. A prolonged hospital stay and thus exposure to the neonatal intensive care unit (NICU) environment could contribute to short- and long-term adverse neurological outcomes. Excessive visual and auditory stimuli, physical and emotional parent–child separation, or disrupted sleep patterns may impact the neonate’s neurological, sensory, and emotional development [3,4,5]. On the one hand, a positive mother–infant interaction requires the absence of mother–infant separation (skin-to-skin contact, positive sensory stimuli, or eye contact) and, on the other hand, a high psychological availability of the mother. If this is missing, it could influence the child’s development [4].

Health professionals have long underestimated the importance of socioemotional development, stress-related brain circuits, and early exposure to pain and discomfort [6]. Moreover, early neurological impairments were first understood to be due to destructive processes in neurons, axons and glial cells, leading to necrotic lesions [7]. With advances in brain imaging techniques, preterm brain lesions were better defined in more recent studies, revealing that the destructive part constitutes a minor component. More specifically, glial progenitors cells respond to white matter damage with an aberrant repair response [8]. Accordingly, the premyelinating cells regenerate and expand but fail to mature, differentiate, and myelinate. The neurocognitive deficits observed in preterm children in the medium and long term are potentially related to these neuronal/axonal disorders [8]. The better understanding of the dysmaturation involving both white and grey matter in the preterm brain has led to the consideration of early interventions that could enhance early brain maturation and ultimately promote neurodevelopment [9]. Indeed, preterm neonates whose mothers had received stress reduction training displayed better maturation and connectivity within the white matter [10].

Developmental care, introduced in the 1990s in the NICU, follows two main goals; to adapt the environment to decrease the neonate’s stress and to reduce the emotional consequences of the prematurity on the family [11]. More specifically, developmental care interventions introduced early during the NICU stay aim to reduce harmful external stimuli, improve the positioning of the neonate (swaddling), strengthen the parent’s role in caring for their child with individualised approaches, such as skin-to-skin contact and massage therapy [6].

We conducted a randomised controlled trial examining the effects of an early intervention, the *Joint Observation* in the NICU (JOIN), delivered in the NICU with the mother and her preterm neonate. Using video feedback, competences of the preterm neonate and reciprocal interactions were demonstrated to the mother [12]. The primary outcome of this study was the difference in maternal perceived self-efficacy between the two groups. The main objective of the present work was to measure the impact of the JOIN intervention on the neurodevelopment of the preterm infant at six months corrected age (CA) in the intervention group compared to the control group. We hypothesised that by reinforcing the perception of parental efficacy, the JOIN intervention would allow for better stimulation of the infants and positively impact their neurodevelopment as measured by a standardised assessment at six months CA [12].

## 2. Materials and Methods

The study protocol, which the ethical committee of the canton of Vaud approved, was previously published [12]. The specific methods relative to the secondary outcome examined in the present study are summarised hereafter. This randomised controlled trial (RCT) was registered on clinicatrials.gov (NCT02736136). This manuscript follows the CONSORT 2010 guidelines [13]. The study was performed in accordance with the Declaration of Helsinki.

### 2.1. Participants

The initial sample involved a population of mothers of preterm neonates born between 28 and 32 6/7 weeks of gestational age (GA), aged less than eight weeks of life, and hospitalised in the Level III NICU of the Lausanne University Hospital, Switzerland. In the case of twin preterm neonates, only the firstborn or the most stable one was included. Exclusion criteria were mothers requiring acute care, under 18 years of age, with an established intellectual disability or psychotic illness, with insufficient French language skills, or mothers of preterm neonates with cardiorespiratory instability (severe brady-apnoea syndrome and oxygen requirement >30%).

### 2.2. Trial Design, Procedure, Data Collection, and Timing

The present study was part of a monocentric randomised controlled (1:1 ratio) interventional study. The randomisation was processed by a computer-generated list of random blocks (https://www.sealedenvelope.com/simple-randomiser, accessed on 15 March 2016) and sealed within numbered envelopes. These envelopes were opened only after the mothers had signed a consent form and completed baseline assessments. The principal investigator and the statistician were unaware of the respective groups the participants had been assigned to. To respect patient confidentiality, all data were coded. Variables regarding pregnancy (including maternal socioeconomic score according to Largo [14]), birth, neonatal morbidity and outcome at 6 months CA were collected.

### 2.3. Intervention and Follow-Up

The mothers assigned to the intervention group were offered to engage in the *joint observation* (JOIN). This interdisciplinary intervention was carried out in two phases; first, a 30 min video recording of care given by the mother to her neonate (e.g., diaper change) and accompanied by a neonatal nurse. In the second phase, which lasted 60 min, the mother watched the video with a child psychologist/psychiatrist and a trained nurse, who offered interactive guidance and video feedback in which mother–infant interactions were observed and analysed. This last step aimed to show to the mother the quality of the parent–infant relationship, the adjustments and reciprocity of the neonate’s and mother’s responses, and the baby’s self-regulation and relational engagement capacities. All interdisciplinary professionals participated in video feedback and interactive guidance training and were supervised at different stages of the study.

The joint intervention is based on four theoretical models of neonatal and infant development. The first model, developed by Brazelton and Nugent, focuses on the detection of the neonate’s competences and fragilities in order to adapt to the infant’s regulation needs [15]. Second, according to the Als’s synactive model, a self-regulated care plan is recommended to limit overstimulation in the NICU [16]. The third model, Bullinger’s sensorimotor approach, assesses whether newborns are receiving adequate sensory stimulation, and manages any subsequent postural and tonal disturbances [17]. The fourth approach is the interactive guidance, which uses video recording of the parent–infant interaction. The video feedback permits one to identify the competences and resources of the parent and to reveal subtle infant cues during usual care. Based on previous research, the interactive guidance through video feedback increases parental sensitivity, while decreasing the stress generated by the NICU stay, and has positive effects on the quality of the mother–infant interaction [18]. For more details, see [12]. 

The mothers in the control group received usual standard care, which included strategies to adapt the NICU environment to the neonate’s needs according to the developmental care principles, promote the parent–infant relationship’s construction, and support parental mental health. All infants at six months CA were invited for a follow-up visit. A developmental paediatrician performed a neurological examination and standardised developmental tests. In addition, a 10 min mother–child play interaction was filmed.

### 2.4. Outcome Measures 

The main outcome of this study concerned the six-month CA neurodevelopment of the preterm neonates, which was evaluated by the Bayley Scales of Infant and Toddler Development, 3rd Edition (BSID-III) [19,20,21]. This standardised test examines three domains of development: cognition, language (receptive and expressive communication), and motor skills (fine and gross motor). The raw scores are converted into scaled scores, which allow the comparison of the child’s performance against age-specific norms. For a subset of participants, the Griffiths Mental Development Scales [22] were also used to assess the six-month CA neurodevelopment.

### 2.5. Statistical Analysis

Baseline variables, including demographic, maternal, perinatal, and postnatal variables, were described with descriptive statistics and compared between the two groups. Outcome variables comprising neurodevelopmental scores, clinical, and growth characteristics were collected at six months CA for each infant. 

SPSS software (version 27) allowed us to analyse the categorical and continuous variables. Categorical variables were described with frequencies and continuous variables with the median and interquartile range. The abnormal distribution was assessed with the Shapiro–Wilk test, which led us to use the Mann–Whitney U test for the continuous variables. For the categorical variables, the *x*^2^ tests of independence were performed. *p*-values below 0.05 were considered statistically significant.

## 3. Results

### 3.1. Population

**Figure 1:**
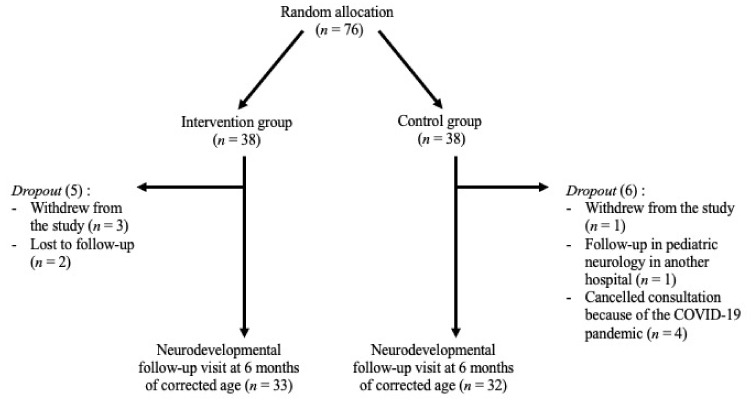
Flow chart of the study.

A population of *n* = 1964 mothers and preterm neonates born between 28 and 32 6/7 weeks of gestation (GA) and less than eight weeks of age were screened for eligibility between March 2016 and February 2020, and *n* = 76 mother–preterm neonate dyads were finally recruited. Five preterm neonates in the intervention group and six in the control group were not seen at the follow-up neurological assessment at six months CA and were therefore excluded from the analyses of the secondary outcome. Thus, neurodevelopmental outcome data were available for a final sample of *n* = 65 subjects. The median (IQR) GA was 30.1 (28.7–31.4) weeks, and the median birth weight was 1240 (990–1456.5) grams in the intervention group and 30.2 (29.6–31.4) weeks and 1237.5 (1006–1523.8) grams in the control group, respectively. The flowchart can be found in Figure 1.

### 3.2. Baseline Characteristics

Baseline characteristics, i.e., demographic, maternal, pregnancy, perinatal, and neonatal, did not differ between the intervention and the control groups (all *p* > 0.05, see Table 1). There were no significant differences between the two groups regarding neonatal morbidity, including brain damage or severity of illness. As for the mothers, there was no single mother in either group, and the maternal education level was not statistically different between the two groups, according to the Largo score.

### 3.3. Six-Month Infant Developmental Outcome

The median age at the follow-up visit was 6 [6,7] months CA in both groups. There was no significant difference in demographic, clinical and growth variables between the two groups. Neurological examination revealed mild anomalies in a small number of infants, including probable transient tone anomalies and mild neurosensory deficits (vision, hearing). Physiotherapy was administered to 15 out of 33 children (45.5%) in the intervention group versus 10 out of 32 children (31.3%) in the control group (*p* = 0.310). Regarding motor milestones, *n* = 27 (81.8%) children in the intervention group and *n* = 29 (90.6%) in the control group demonstrated a stable sitting position (*p* = 0.475).

The subset of children tested with the BSID-III comprised *n* = 20 participants in the intervention and *n* = 16 in the control groups. As described in Table 2, there was no statistically significant difference in the standardised developmental tests between the two groups. Two of the sixty-five children could not be scored with the Bayley or Griffiths scales for technical reasons. Four children (two children in each group) in the Bayley motor subscale and three children (two children in the intervention group and one in the control group) in the cognitive subscale scored >115 (maximum = 124). Four children (three children in the intervention group and one in the control group) in the Bayley motor subscale scored <85 (minimum = 73). For the Griffiths scales, only one patient in the control group had a global score between 70 and 85, classified as moderately abnormal. The results are summarised in Table 2. There were no unintended or adverse effects associated with this intervention.

## 4. Discussion

Maternal stress, early mother–infant separation, and environmental factors in the NICU may negatively influence the infant’s development. A positive mother–infant interaction could play an essential role in mitigating the relationship between prematurity and potential adverse neurodevelopmental outcomes. We hypothesised that an early intervention supporting the mother–infant relationship might indirectly improve infant development. We examined the effects of the JOIN intervention delivered by an interdisciplinary team in the NICU on neurodevelopmental outcomes. As reflected by the Bayley and Griffiths scores at the corrected age of six months, neither the mental nor the psychomotor development indices differed significantly between the control and intervention groups.

In response to the potentially harmful effects of the neonatal environment, the concept of “developmental care” was developed 30 years ago. In some cases, it refers to several simple measures (noise reduction, light reduction, positioning, non-nutritive sucking, or skin-to-skin contact) [6,23]. Others may refer to more comprehensive programs, such as the Neonatal Individualized Developmental Care Assessment Program (NIDCAP), Infant Behavioral Assessment and Intervention Program (IBAIP), Mother–Infant Transaction Program (MITP), or the Family Nurture Intervention in the Neonatal Intensive Care Unit (FNI) [23,24,25,26]. Milgrom et al. presented the modified MITP-M, an intervention repeated once a week for nine weeks in the NICU and once at home, which resulted in improved language and cognitive skills at six months in the intervention group [23,25]. Welch et al. studied the FNI, another intervention involving support in touching the child, encouraging mothers to talk to children about their feelings, and making eye contact as often as possible [24]. At 18 months CA, FNI infants showed improved cognitive and language competences [24]. Although the JOIN intervention did not demonstrate comparable positive results on the child’s neurodevelopment, the intervention was positively received by the mothers and the interdisciplinary team (results not reported here).

Several factors can explain the results of our study. First, the targeted population seemed to be very homogeneous. In most cases, the mothers appeared to be well educated, lived in a supportive environment, and were followed up by a paediatrician and/or therapist (physiotherapist). As for the children, the neonatal morbidity was of either mild or moderate severity during the NICU stay. In particular, there were few children with moderate or severe bronchopulmonary dysplasia, a known risk factor for the occurrence of neurodevelopmental disorders. Similarly, severe brain damage was not observed in this population. Although not significant, the length of stay of neonates in the intervention group was 12 days longer than in the control group, suggesting that professionals continued to provide psychological support and counselling to the parents, which probably reduced the additional contribution of JOIN. Thus, this intervention did not provide more benefits in a context where participants in both groups were well supported. Previous studies have suggested maternal depression and social disadvantages represent an increased risk of adverse neurodevelopment in preterm children [27]. The implementation of this intervention in more vulnerable populations with less support and education might be more effective, allowing us to determine if it could be generalised. The intervention would then need to be tested in other centres or in areas with a different socioeconomic index.

Second, neurodevelopmental assessment was performed at six months CA, which might be too early to capture the subtle cognitive, language, and motor development differences. Results of the standardised tests showed very little variability, with the vast majority of children having scores considered as normal [20,21]. Additionally, Nordhov et al. showed better cognitive performance at five years of age after being exposed to the MITP in the neonatal period [28]. Similarly, longer-term assessment with comprehensive neuropsychological evaluation might unveil differences between the JOIN and the control groups. Third, a repeated long-term intervention over time could have had more lasting effects. Milgrom et al. examined the effect on neurodevelopment of enhanced MITP delivered over a more extended period in the NICU, i.e., weekly sessions over 9 weeks, followed by a session at home [25]. The results showed that communication skills in the intervention group were more advanced after six months. In the same context, Newnham et al. described a time-repeated modified MITP (up to three months) that resulted in higher communication scores at two years [29]. Fourth, the video guidance used in JOIN was intended to support the mother directly, but with only putative indirect effects on the neonate’s brain development [30]. Yet, the measured neurodevelopmental outcome at six months may not be as appropriate as other outcomes, such as the children’s socioemotional competences. For instance, in the Victorian Infant Brain Studies (VIBeS Plus), an interdisciplinary intervention consisting of visiting parents in their homes to educate them about child self-regulation and parent-child relationships resulted in less prevalent externalised and dysregulated behaviour in children at 24 months of CA [31]. Additionally, other studies showed that the socioemotional deficits were even more prevalent in infants of mothers experiencing mental health disorders, such as depression and anxiety [27,32]. Hence, studying another outcome, such as the ability to understand and appropriately display emotional and social responses or behaviours, could have revealed more impact of the JOIN intervention. In contrast, while considering the absence of a significant positive impact on the neurodevelopment, infants in the control group or more generally those whose mothers are not able to participate in such specialised care due to other life-stressors, would not have missed the presumed benefits of this intervention.

Despite the study’s robust methodological conduct, some limitations can be noted. Neurodevelopmental assessment was performed using two different tests (Griffiths scales and BSID-III), whose scores are not directly interchangeable [33]. This approach resulted in a smaller sample size in each subgroup and decreased statistical power. It should also be noted that the intervention was performed by an interdisciplinary group of caregivers, which led to a certain level of heterogeneity in how the intervention was carried out, although all professionals had been trained and were supervised.

### Perspectives

It is possible that modifying the intervention, for instance by repeating once a month until the neonate reaches 3 months CA whether in the hospital or at home, could significantly increase the effect of the *joint observation* by offering a more prolonged support to the family. It would also be interesting to investigate the effects of this intervention on a targeted, more vulnerable subgroup, such as very preterm infants whose mothers have a history of mental health problems or who experience the birth of their very preterm infant as particularly stressful or even traumatic.

Further studies may need to measure broader aspects of development, including socioemotional competences and self-regulation behaviour. In addition, for a family-centred care unit to succeed, future studies should test interventions involving the father in the mother–father–child triad.

## 5. Conclusions

At six months CA, the *joint observation* (JOIN) performed in the NICU in the early neonatal period did not improve the neurodevelopment of preterm neonates. The intervention was part of a more complete developmental care program supporting the parents and their neonate, who faced several challenges that could negatively impact their relationship, with long-term consequences. As preterm infants are at risk of neurodevelopmental impairment, early interventions are of utmost importance, focusing on the parent–infant interaction, which can mediate the relationship between prematurity/NICU environment and the neurodevelopment. Future research should investigate whether, with individualised tailoring, integrating the JOIN into standard care might help strengthen parenting and attachment skills, improve mother–child interaction, and subsequently enhance the preterm infant’s neurodevelopment.

## Figures and Tables

**Table 1:** Pregnancy and neonatal variables.

	Intervention Group (N = 33)	Control Group (N = 32)	*p*-Value
**Pregnancy variables**			
Maternal age (M, IQR)	32 (30–34.5)	32.5 (30–36.8)	*p* = 0.381 U = 461.5
Number of pregnancies (M, IQR)	1 (1–2)	1 (1–2)	*p* = 0.810 U = 512
Maternal Largo score (M, IQR) missing *n* = 1	2 (2–3.8)	2 (1–3)	*p* = 0.319 U = 441
Paternal Largo score (M, IQR) missing *n* = 7	3 (2–4)	3 (1.5–3)	*p* = 0.802 U = 405
Multiple pregnancy (N, %)	9 (27.3)	5 (15.6)	0.367
Assisted reproduction (N, %)	4 (12.1)	3 (9.4)	1
Prenatal steroids (N, %)	26 (78.8)	29 (90.6)	0.303
**Neonatal variables**			
Female (N, %)	21 (63.6)	16 (50)	0.321
Gestational age, weeks (M, IQR)	30.1 (28.7–31.4)	30.2 (29.6–31.4)	*p* = 0.412 U = 465.5
Birth weight, grams (M, IQR)	1240 (990–1456.5)	1237.5 (1006–1523.8)	*p* = 0.896 U = 518
Small for gestational age (N, %)	5 (15.2)	7 (21.9)	0.562
Head circumference at birth, centimeters (M, IQR)	26.5 (25–28.3)	27 (25.6–28.5)	*p* = 0.429 U = 468
Inborn (N, %)	31 (93.9)	29 (90.6)	0.672
Arterial cord pH (M, IQR) missing *n* = 18	7.3 (7.2–7.3)	7.3 (7.2–7.3)	*p* = 0.676 U = 253.5
Apgar 5 min (M, IQR)	9 (8–10)	9 (8–9)	*p* = 0.325 U = 456
CRIB score (M, IQR)	6 (3.5–7)	5 (3–6.8)	*p* = 0.500 U = 477
Mechanical ventilation, hours (M, IQR)	0 (0–13)	0 (0–9.5)	*p* = 0.510 U = 484.5
Noninvasive ventilation, hours (M, IQR) missing *n* = 1	658.5 (229.5–919.8)	304.5 (65.3–789.8)	*p* = 0.093 U = 387
Moderate and severe BPD – bronchopulmonary dysplasia (N, %)	2 (6.1)	3 (9.4)	*p* = 0.443
Postnatal steroids (N, %)	2 (6.1)	2 (6.3)	1
Persistent ductus arteriosus (PDA) (N. %)	33 (100)	32 (100)	0.303
- medical treatment	3 (9.1)	6 (18.8)	
- surgical ligature	0 (100)	0 (100)	
Other surgery (N, %)	1 (3)	3 (9.4)	0.355
Early onset sepsis (EOS) (N, %)	1 (3)	2 (6.3)	0.613
Late onset sepsis (LOS) (N, %)	7 (21.2)	6 (18.8)	1
Necrotizing enterocolitis (NEC) (N, %)	1 (3)	0 (0)	1
Intraventricular hemorrhage (IVH) (N, %)			0.514
- grade 1	4 (12.1)	7 (21.9)	
- grade 2	1 (3)	1 (3.1)	
- grade 3	0 (0)	1 (3.1)	
- grade 4	0 (0)	0 (0)	
Periventricular leukomalacia (PVL) (N, %)	33 (100)	32 (100)	0.513
- grade 1	1 (3)	2 (6.3)	
- grade 2	1 (3)	0 (0)	
- grade 3	0 (0)	0 (0)	
- grade 4	0 (0)	0 (0)	
Retinopathy of prematurity (ROP) (N, %)	1 (3)	0 (0)	1
Abnormal hearing test (N, %)	1 (3)	1 (3.1)	1
Length of stay in level III hospital, days (M, IQR)	49 (36 – 62)	37 (21 – 56)	*p* = 0.198 U = 430
Issue (N, %)	33 (100)	32 (100)	0.136
death	0 (0)	0 (0)	
transfer to another unit/hospital	15 (45.5)	21 (65.6)	
discharge home	18 (54.5)	11 (34.4)	

IQR: interquartile range, M: median, U: Mann–Whitney U test.

**Table 2:** Six-month CA assessment variables, neurological exam variables and standardised developmental tests.

	Intervention group (N = 33)	Control group (N = 32)	*p*-value
**Six-month CA assessment variables**			
Chronological age, months (M, IQR)	9 (8–9)	8 (8–9)	*p* = 0.346 U = 461.5
Corrected age, months (M, IQR)	6 (6–7)	6 (6–7)	*p* = 0.447 U = 476.5
Hospitalization after NICU discharge (N, %)	7 (21.2)	8 (25)	0.775
Relevant medical condition (N, %)	7 (21.2)	7 (21.9)	1
Physiotherapy (N, %)	15 (45.5)	10 (31.3)	0.310
Daycare attendance (N, %) missing *n* = 2	4 (12.1)	3 (10)	0.646
Maternal return to work (N, %) missing *n* = 3	23 (69.7)	18 (62.1)	0.596
Siblings (N, %) missing *n* = 1	13 (40.6)	13 (40.6)	1
Sleep disorder (N, %)	0 (0)	2 (6.3)	0.238
Acquisition of the sitting position (N, %)	27 (81.8)	29 (90.6)	0.475
Acquisition of moving on 4 points (N. %)	12 (36.4)	12 (37.5)	1
Weight at 6 months, grams (M, IQR)	7400 (6600–8020)	7175 (6362.5–8462.5)	*p* = 1 U = 528
Length at 6 months, centimeters (M, IQR)	68 (65–69)	67 (64–69.8)	*p* = 0.721 U = 501
Head circumference at 6 months, centimeters (M, IQR)	43.2 (42–44.2)	44 (42.8–44.5)	*p* = 0.076 U = 393
**Neurological exam variables**			
Tone disorder (N, %)	4 (12.1)	6 (18.8)	0.511
Visual impairment (N, %)	2 (6.1)	2 (6.3)	1
- strabismus	1 (3)	1 (3.1)	
- retinal immaturity	0 (0)	1 (3.1)	
- retinal detachment and retinopathy of prematurity	1 (3)	0 (0)	
Hearing impairment: abnormal auditory evoked potentials (N, %)	1 (3)	0 (0)	1
**Standardised developmental tests**			
Bayley—cognition scale (M, IQR)	110 (105–115) *n* = 20	115 (106.3–115) *n* = 16	*p* = 0.102 U = 110.5
Bayley—language scale (M, IQR)	94 (91–100) *n* = 20	97 (91.8–102.3) *n* = 16	*p* = 0.479 U =138
Bayley—motor scale (M, IQR)	97 (85–106) *n* = 20	100 (91.8–111.5) *n* = 16	*p* = 0.315 U = 128.5
Griffiths—developmental quotient (M, IQR)	99 (96.3–110.8) *n* = 12	100 (92–103) *n* = 15	*p* = 0.591 U = 79

IQR: interquartile range, M: median, U: Mann–Whitney U test.

## Data Availability

Not applicable.
